# The Role of Sphingolipids in Allergic Disorders

**DOI:** 10.3389/falgy.2021.675557

**Published:** 2021-06-14

**Authors:** Araceli Díaz-Perales, Maria M. Escribese, María Garrido-Arandia, David Obeso, Elena Izquierdo-Alvarez, Jaime Tome-Amat, Domingo Barber

**Affiliations:** ^1^Centro de Biotecnología y Genómica de Plantas (CBGP), Universidad Politécnica de Madrid (UPM), Instituto Nacional de Investigación y Tecnología Agraria y Alimentaria (INIA), Madrid, Spain; ^2^Basic Medical Sciences Department, Facultad de Medicina, Instituto de Medicina Molecular Aplicada (IMMA), Universidad San Pablo CEU, CEU Universities, Madrid, Spain; ^3^Centro de Excelencia en Metabolómica y Bioanálisis (CEMBIO), Facultad de Farmacia, Universidad San Pablo CEU, CEU Universities, Madrid, Spain

**Keywords:** sphingolipid, inflammation, allergy, sphigosine-1-P, ceramide-1-P, disruption epithelial barrier

## Abstract

Allergy is defined as a complex chronic inflammatory condition in which genetic and environmental factors are implicated. Sphingolipids are involved in multiple biological functions, from cell membrane components to critical signaling molecules. To date, sphingolipids have been studied in different human pathologies such as neurological disorders, cancer, autoimmunity, and infections. Sphingolipid metabolites, in particular, ceramide and sphingosine-1-phosphate (S1P), regulate a diverse range of cellular processes that are important in immunity and inflammation. Moreover, variations in the sphingolipid concentrations have been strongly associated with allergic diseases. This review will focus on the role of sphingolipids in the development of allergic sensitization and allergic inflammation through the activation of immune cells resident in tissues, as well as their role in barrier remodeling and anaphylaxis. The knowledge gained in this emerging field will help to develop new therapeutic options for allergic disorders.

## Introduction

Allergic disorders, including asthma, atopic dermatitis (AD), and food allergy (FA), entail significant morbidity worldwide. These pathologies are type 2 (T2) immunological diseases that result from complex mechanisms that include aspects such as genetic background, epigenetic background, and environmental influences (1). They show distinctively diverse clinical manifestations, suggesting the presence of additional unique pathogenic processes involved in shaping their heterogeneous phenotypic attributes (2).

The prevalence of allergic diseases is around 25% of the population of the world, with an increasing trend, regardless of age, gender, or ethnicity. The treatment of these diseases is based on the identification of their sources (allergens) and their avoidance, which is not always possible (2). Allergic patients with cross-reactivity, sensitization to multiple allergens, require in most cases prophylactic and symptomatic treatments. Current treatments are often suboptimal, resulting in a reduced quality of life for the patients and placing an economic burden on society. A deeper understanding of their pathogenic mechanisms might lead to much needed and improved disease biomarkers (3).

Recently, it has been proposed that sphingolipids and the enzymes involved in their metabolism may play a role in the development of allergic diseases (4, 5). Sphingolipids are key factors in cell growth, survival, inflammation, and tissue remodeling (4–8). Among them, sphingosine-1-phosphate (S1P) is of particular interest due to its pro-inflammatory effects (4, 5); however, other sphingolipids such as ceramide (CER) have the opposite effect. Therefore, the balance between sphingolipid synthesis and degradation is crucial for the regulation of inflammation and tissue remodeling (4, 5).

In this review, we summarize the biochemistry of sphingolipids, as well as their immunological importance and their role in the pathogenesis of allergic disorders.

## Sphingolipids

Sphingolipids and their intermediates are an extraordinarily diverse group of molecules with a vast array of physical properties, which are present in almost all eukaryotic organisms (9). Their basic structure is composed of a sphingoid base backbone attached by an amide bond to different fatty acids or a head group at the primary hydroxyl (3). The nature of the head group is highly variable, and it defines the different sphingolipid species: CER, in which the head group is substituted by hydrogen; sphingomyelins, in which the head group is substituted by phosphocholine; and cerebrosides or gangliosides, in which hundreds of different monosaccharides in a single or combined form make the compound (10, 11).

**Figure 1:**
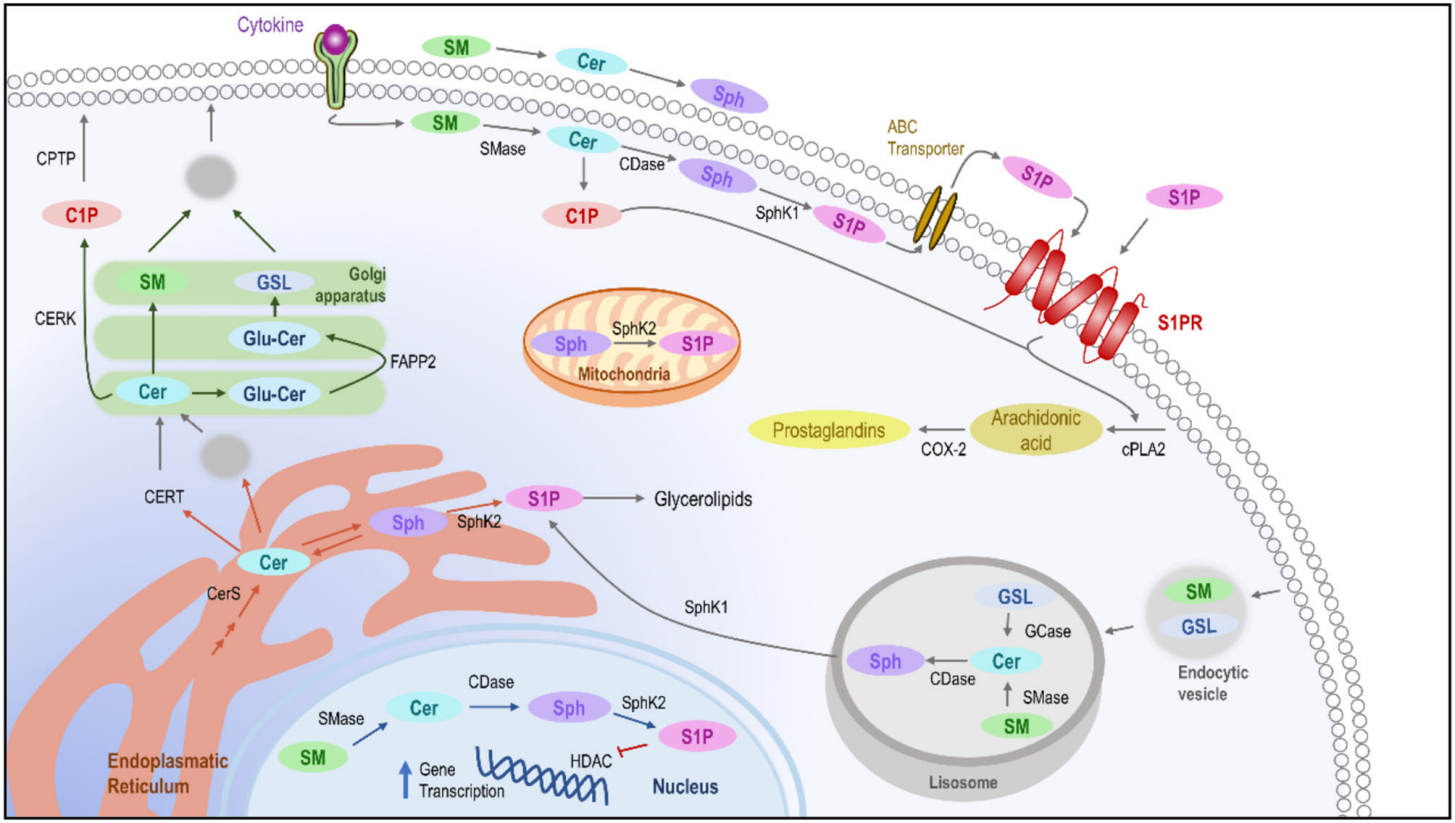
Intracellular pathways of sphingolipids. *De novo* ceramide synthesis takes place in the ER where it might be delivered to the Golgi apparatus using the ceramide transfer protein by vesicular transport to synthesize sphingomyelin glucosylceramide or C1P, which will become part of the plasmatic membrane. In the membrane, after the presence of stimuli, the different enzymes implicated in the homeostasis of the sphingolipids are activated, leading to the production of C1P and S1P, which in the end leads to the activation of phospholipase-A2 and the production of prostaglandins as inflammatory mediators. Moreover, the sphingolipids can also be metabolized in lysosomes, leading to the release of sphingosine and in the cellular nucleus, where S1P could block the transcription of genes related to inflammation. ER, Endoplasmic reticulum; C1P, Ceramide-1-Phosphate; CDase, Ceramidase; CER, Ceramide; CERK, Ceramide kinase; CERS, Ceramide Synthase; CERT, Ceramide transfer protein; CPTP, Ceramide-1-phosphate protein transport; GSL, Glucosylceramide; SM, Sphingomyelin; SMase, Sphingomyelinase; S1P, sphingosine-1-phosphate; SPH, Sphingosine; SphK, Sphingosine kinase.

The simplest member, CER, is often referred to as the core of sphingolipid metabolism because of its fundamental role in the accumulation of *de novo* sphingolipids as well as in serving as a precursor for derived molecules. The biosynthesis of these lipids involves multiple enzymatic processes for the conversion to different species, where each enzyme serves as a control point for maintaining homeostasis (12) (Figure 1).

From a biological point of view, sphingolipids are found mainly in the plasma membrane, the lumen of intracellular organelles, and lipoproteins, where they act as a reservoir of bioactive metabolites for functions related to signaling, cell survival and growth, immune cell trafficking, and vascular and mucosal integrity (8, 13, 14). Metabolites such as S1P and ceramide-1-phosphate (C1P) are the most common and potent bioactive mediators (15). An increase in C1P along with a decrease in S1P induces cell death pathways, while the opposite scenario results in the stimulation of anti-apoptotic pathways (8, 12, 15–18). C1P is related to caspase-3-mediated apoptosis and cell cycle arrest while S1P is related to proliferation and survival (19, 20).

### Sphingosine-1-Phosphate

Sphingosine-1-phosphate is involved in the differentiation of many immune cell types, inducing changes in their functional phenotypes and taking part in the regulation of pro-inflammatory cytokines and eicosanoids. Simultaneously, specific stimuli may cause the activation of intracellular kinases to produce higher amounts of S1P. In the case of cells circulating through the vascular system, the presence of S1P in the plasma does not seem to have an effect on them *per se*, suggesting that the regulation of SIP receptors (S1PRs) on the membrane, rather than the presence of S1P, is the main factor mediating their effects (8, 16, 21).

Maintained concentrations of S1P in plasma and tissues within a specific range are essential for barrier function. Physiologic S1P plasma concentrations (0.5–1 μM) keep microvascular barrier integrity *via* ligation to the S1PR (22). In studies in the animal model, exogenous addition of S1P to endothelial cells increased monolayer integrity rapidly and dose-dependently through the same receptor.

**Figure 2:**
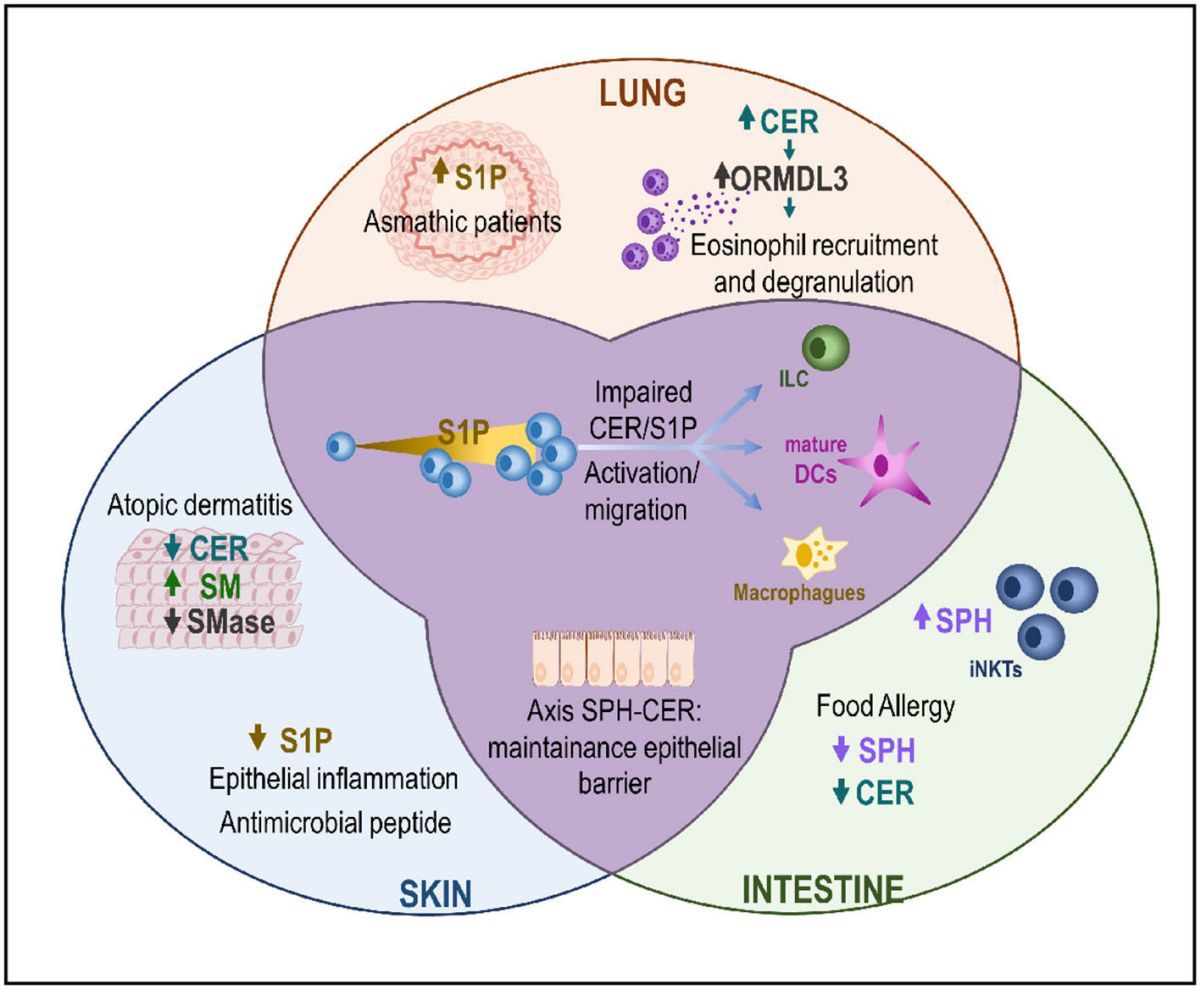
The CER/S1P axis in the MALT. Changes in the CER/S1P ratio triggers activation and migration pathways in ILCs, dendritic cells, and macrophages. This axis plays a role in different compartments of the MALT (skin, lung, and/or intestine) with different organ-dependent results. In the skin, downregulation of CER and SM causes increased permeability, which is associated with a higher penetration of bacteria and/or allergens, which in turn will increase inflammation. In the lung, asthmatic patients show higher levels of S1P. This disease is tightly related to increased ORMDL3 expression, with a concomitant increase in the CER levels and eosinophilia. Finally, in the intestines, decreased levels of sphingolipids and S1P are associated with the development of FA in patients. CER, Ceramide; DC, Dendritic cells; ILC, Innate lymphoid cells; iNKTs, invariant natural killer T cells; SM, Sphingomyelin; SMase, Sphingomyelinase; S1P, sphingosine-1-phosphate; SPH, Sphingosine; ORMDL3, Orosomucoid-like 3; FA, Food allergy.

In addition, S1P regulates the activation and functions of many immune cells and regulates diverse immunological processes (14, 23, 24). For example, it acts as a chemoattracting signal for macrophages and mast cells (MCs), leading them to inflammation sites for tissue repair (25, 26) (Figure 2).

In the case of S1P receptors, five members of this family (S1PR1–S1PR5) have been described to bind S1P with high affinity (27) (Table 1). They belong to the G-protein coupled family and are expressed widely but differently depending on the cell type or organ. The S1P–S1PR axis regulates the traffic and migration of most immune cell types, cytoskeleton rearrangements, cell-matrix contacts, adhesive junction assembly, and barrier integrity (16, 21).

**Table 1:** An overview of sphingosine-1-phosphate (S1P) receptors.

**Receptor**	**Tissue**	**Immune cell type**	**Function**	**Location**
**S1PR1**	Endothelial cells Muscle cells Fibroblasts Melanocytes	Macrophages Neutrophils Eosinophils Dendritic cells Lymphocytes	Maintenance of vascular barrier function Cell migration through a S1P gradient Inhibits angiogenesis Required for normal embryonic heart development and normal cardiac morphogenesis.	Endosome Plasma membrane
**S1PR2**	Mainly in vascular endothelium Low tissue specificity	Monocytes Macrophages Basophil Eosinophils Mast cells Lymphocytes	Antagonistic role with S1PR1 in macrophages, repulsing them from S1P. Regulates macrophages phagocytic capacity Cell proliferation Suppression of apoptosis	Plasma membrane
**S1PR3**	Low tissue specificity	Different immune cells Cell enriched: monocytes	Dendritic cell maturation Macrophage chemotaxis and reactive oxygen species production Neutrophil and eosinophil recruitment Contributes to the regulation of angiogenesis and vascular endothelial cell function	Plasma membrane
**S1PR4**	Blood Bone marrow Lymphoid tissue	Dendritic cells, T cells B cells Granulocytes	Regulates the ability of dendritic cells to migrate	Membrane Mitochondria
**S1PR5**	Blood Brain Skin	Oligodendrocytes Eosinophils Mast cells NK cells T cells	Induces the egress of immune cells in the bone marrow	Intracellular Membrane

S1PR1 expression was first described in the endothelium (28). This receptor is necessary for the maintenance of the vascular barrier function, and it is tightly related to its permeability. It is also expressed in immune cells such as macrophages, neutrophils, eosinophils, dendritic cells, and lymphocytes, where it plays a role in migration, through an S1P gradient. Furthermore, it is related to the activation of PI3K and PKC pathways (29).

S1PR2 is also expressed in the vascular endothelium and in monocytes, macrophages, eosinophils, MCs, and lymphocytes. In macrophages, it has an antagonistic role to S1PR1, and it regulates the phagocytic capacity as well (25). Besides this, it activates the Rho/ROCK signaling pathway and has a pro-inflammatory role due to the activation of the NF-κB transcription factor (30).

S1PR3 is expressed in different immune cells, having a role in dendritic cell maturation, macrophage chemotaxis, and reactive oxygen species production, and neutrophil and eosinophil recruitment (25). S1PR4 antagonizes with S1PR1 in dendritic cells, regulating their ability to migrate from the periphery after antigen uptake. It also activates Rho-kinases and promotes cytoskeletal rearrangement (31). S1PR5 is mainly found in oligodendrocytes, eosinophils, and MCs. It induces the egress of immune cells in the bone marrow (25).

### Ceramide-1-Phosphate

Ceramide-1-phosphate, produced in the trans-Golgi by CER-kinase, activates cytosolic phospholipase-A2α, the enzyme that releases the eicosanoid precursor arachidonate (32), and also increases the regulation of another family of transcription factors closely related to the induction of cyclooxygenase-2 and the subsequent production of eicosanoids (15). Moreover, C1P can mediate MCs degranulation in a Ca^2+^ dependent manner. Ceramide kinase (CERK) required Ca^2^+ for its activation, it being calmodulin the probable Ca^2+^ sensor (33). As degranulation involves the fusion of vesicles with the plasma membrane, it has been suggested that the conversion of CER to C1P may alter the equilibrium of the sphingolipids in the membrane and lead to further fusion of the vesicles (33). In contrast, C1P has been shown to inhibit the TNF release (34, 35).

While many aspects of the C1P receptor remain unknown, it has been described that the extracellular recognition of C1P induces the activation of the CERK pathway, causing a mitogenic effect in macrophages (17), as it promotes cellular proliferation and growth. Inside the cells, this molecule regulates the interaction of phospholipase-A2α with its inhibitors, leading to the inflammatory response mediated by arachidonic acid and prostaglandins (36).

## Sphingolipids and Allergic Disorders

### Sphingolipids and Asthma

Asthma is a heterogeneous pathology influenced by both environmental and genetic factors. It is clinically defined as a chronic lung disease associated with narrowing of the airways, bronchial hyperactivity, and increased mucus production. Sphingolipids are the major components of lung surfactants, and they are essential for the maintenance of epithelial barrier integrity (37).

Sphingolipid metabolites, especially S1P, contribute to the pathogenesis of asthma. In the middle of the last century, it was described that the secretions of patients with respiratory diseases, such as cystic fibrosis or viral infections, showed higher lipid content compared to controls (38). In the same way, levels of S1P were higher in the lungs of patients with allergic asthma (39); significantly, in bronchoalveolar lavage fluid (BALF) from asthmatic patients after allergen challenge (40).

Experimental models of allergic asthma have demonstrated that S1P plays a crucial role in the development of the asthmatic phenotype (39, 41). Subcutaneous injections of S1P resulted in the progressive development of airway inflammation and air hyperreactivity response (AHR), which reached their maximum 2 weeks after the last dose (39). Moreover, local application of exogenous S1P in a murine model aggravated the allergen-induced airway inflammation, resulting in an increased contraction of the bronchi and increased airway resistance, as well as MC and eosinophil recruitment in the lung (41, 42).

On the contrary, inhibition of sphingosine kinase (SphK) 1, which is crucial for the synthesis of S1P, resulted in attenuated airway inflammation and AHR in a model of allergic asthma (43). Similarly, application of the immunosuppressant drug FTY720/fingolimod (sphingosine analog), which interferes with S1P–S1PR interaction, inhibited the airway remodeling, which followed a repeated allergen exposure in rats (44).

Ceramide synthesis also seems to be involved in asthma, based on the increased expression of Orosomucoid-like 3 (ORMDL3), an endoplasmic reticulum (ER)-resident transmembrane protein that regulates the activity of serine palmitoyltransferase (SPT) (45). It has also been suggested to be involved in ER-mediated calcium signaling and stress response in immune cells (46); as well as in eosinophil trafficking, recruitment, and degranulation in murine models of allergic asthma (47).

This protein acts as a negative regulator of sphingolipid biosynthesis. Its overexpression in the respiratory tract of asthmatic patients promotes CER synthesis and the development of T2 responses in the airways (45, 48). The nasal administration of FTY720/fingolimod reduced ORMDL3 expression and CER levels, mitigating the airway inflammation and mucus hypersecretion in house dust mite–challenged mice (49).

Polymorphisms at ORMDL3 locus have been associated with increased risk for asthma (50–52), severe asthma (53, 54), and early viral respiratory infections (55), and it is a well-known risk factor for persistent wheezing and a risk factor for asthma development (55, 56). In this way, variants at the 17q21 locus have been shown to enhance the association between early respiratory infections and childhood asthma (55, 56). In particular, infections with human rhinovirus (HRV), the most common trigger for asthma exacerbations (57), are associated with a more than 10-fold increased odds ratio for childhood asthma in children who carry the asthma-associated ORMDL3 genotype (56).

In mouse lungs, ORMDL3 expression can be increased by a variety of stimuli, such as allergens, tobacco smoke, and lipopolysaccharides (58). Although ORMDL3 polymorphisms have not been associated with atopy (51, 59, 60), some seem related to T helper cell type 2 (Th2) cytokine responses (61) and asthmatic responses to allergens (62). Overexpression of human ORMDL3 in transgenic mice showed an associated increase in airway remodeling (smooth muscle, fibrosis, mucous production) and an enhanced IgE response compared to wild-type mice following allergen challenge (58). It has been demonstrated that there are differences in DNA methylation at the ORMDL3 gene between asthmatics and controls (63).

### Sphingolipid and Atopic Dermatitis

Mammalian skin contains extensive amounts of lipids, mainly in the epidermis, where ceramides, cholesterol, and free fatty acids can be found. Keratinocytes produce sphingolipids *de novo* from palmitoyl-CoA and serine in the form of glucocerebrosides and sphingomyelin. These are secreted into the stratum corneum, the outer layers of the skin, where they are transformed into ceramides for the skin barrier (64). Skin lipids, directly or indirectly, are key components in maintaining the barrier homeostasis, and any defects in the metabolism of skin lipids are related to skin barrier dysfunction (65–67).

Epidermal barrier dysfunction has been observed in both areas with AD lesions and areas without such lesions, in the shape of increased trans-epidermal water loss, raised skin pH, altered surface microbiota colonization pattern, and an affected ceramide profile. The underlying attenuated barrier function in AD results in the continuous generation of cytokines and chemokines, a pro-inflammatory cytokine cascade, and intrusions of allergens and antigens, which contribute to the “atopic march” (68).

The alteration of CER composition in the epidermis not only contributes to an impaired skin barrier but also promotes the development of inflammatory and allergic properties in individuals with AD (69, 70). The content of ceramides is significantly lower in patients with AD with FA than in those without (71) FA. Over the past years, several studies have reported an increase in the expression of sphingomyelin deacylase and a reduction in the expression of sphingomyelinase (SMase) in AD, which may cause a decrease in total ceramides (72). In relation to this, mice lacking the enzyme ceramide synthase 4 (CERS4) present a disrupted epidermis and a CD45-infiltrated dermis, even without the topical application of an allergen or irritant (73). In contrast, high levels of CERS4 in AD contribute to the enhanced synthesis of short-chain ceramides (74), constructing a healthier skin, more resistant to aggressions.

Another role of the sphingolipids in the skin is related to bacterial homeostasis. In this context, an increase in S1P lyase activity ends in a reduction of S1P concentrations (64, 75). Since S1P upregulates cathelicidin, an antimicrobial peptide produced by keratinocytes, its reduction also compromises the first immune response (76). This alteration results in the growth of special microflora of the skin, like *Pseudomonas aeruginosa* and *Staphylococcus aureus*, both of which relate to AD severity (77). The defense system of the skin against bacterial invasion is significantly disrupted in patients with AD. Interestingly, T2 cytokines, interferon (IFN) -γ, and tumor necrosis factor (TNF)-α perpetuate the vicious cycle between the epidermal barrier dysfunction and AD pathogenesis (78).

Finally, not only AD but allergic contact dermatitis is also affected by S1P. In a murine contact dermatitis model, the topical application of S1P reduced the weight and the cell number in regional lymph nodes (79). The number of dendritic cells migrating from the skin to the lymph node was reduced, and the cytokine pattern in the regional lymph nodes was affected by the topical treatment with S1P (80).

### Sphingolipids and Food Allergy

Food allergy has been characterized by a marked decrease in levels of sphingolipids, including sphingomyelins and ceramides, in plasma (81). These changes observed in patients with FA suggest a low ceramide production and attenuation of additional distal steps in the conversion of ceramides into sphingomyelins, possibly at the level of the enzymes sphingomyelin synthase and SMase.

Regarding sphingolipids and protein interactions (i.e., protein carriers), many aspects are still unknown. However, results carried out by incubating peripheral blood mononuclear cells with these sphingolipids suggest that their presence is essential to induce the activation of invariant Natural killer T cells (iNKT) cells, thereby promoting the production of T2 cytokines and facilitating IgE mediated sensitization (82–84). iNKTs can recognize lipid ligands presented by the atypical MHC class I molecule CD1d, including human sphingolipids, as well as *Bacteroides*, plants, and animals. The role of this cell type in the pathogenesis of FA, in particular, and in allergic disorders, in general, remains unknown. Children alleric to cow's milk show a decrease in the number of iNKT cells in the blood, while an increase has been reported after milk desensitization (85).

In the intestine, sphingolipids can be found in large quantities, not only from *de novo* synthesis but mostly because of their abundance in food such as meat, milk, and eggs (86). Sphingolipids from food intake are metabolized to CER and sphingosine (SPH) by the intestinal epithelial cells (86). An excess of sphingolipids in the diet is associated with an increased population of plasma cells producing IgA antibodies in the colon, as well as the development of FA by the expansion and recruitment (87) of intestinal macrophages.

Plants are another source of sphingolipid. Some studies suggest that they constitute up to 10% of plant lipids (88). Curiously, over 50% of all plant allergens are lipid carriers (89), and these ligands may act as adjuvants and tilt the immune system toward a T2 (pro-allergenic) response (82). In the case of allergenic Lipid Transfer Proteins from plants (nsLTPs), their natural ligand is a sphingolipid derivative (83, 84, 90). Recent evidence suggests that this ligand could be a functional analog of human SPH since it interacts with SphK with the concomitant phosphorylation, which could mimic the function of S1P in human inflammatory responses (91).

On the other hand, the skin also may play an important role in FA sensitization. The skin has been classically described as a tolerogenic tissue, given the predisposition of epidermal Langerhans cells to produce IL10 upon stimulation, as well as their inability to translocate NFκB-related transcription factor RelB to the nucleus (92, 93). However, there is growing evidence that environmental stressors can reverse this tolerogenic environment and elicit T2 responses to promote FA sensitization (94). The clearest evidence is the relationship between AD and FA: more than 80% of patients with FA have or have had AD (95). On the other hand, systemization of immune reactions initiated locally in the skin has been reported not only in FA but also in defense responses against *S. aureus*, proving that epicutaneous exposure to pathogenic bacteria ameliorates the symptomatology of secondary infections in the lung through an IgE- and mastocyte-mediated mechanism (96).

The mechanism by which the damage response in the skin can be dispersed throughout the body remains to be detailed. However, it seems increasingly clear that there is a role for invariant lymphoid cells (ILC) 2 in sensitization spreading, favoring the appearance of symptoms at other sites in the mucosa, such as the gastrointestinal tract. ILCs are immune cells of lymphoid origin that functionally resemble T cells but lack specific antigen receptors. They quickly respond to alterations of tissue homeostasis producing multiple cytokines that are important for the induction and regulation of inflammation. They are found in both lymphoid and non-lymphoid tissues, being particularly abundant on the mucosal surfaces of the intestine, lung, and skin, while they are infrequently detected in peripheral blood (97, 98).

Invariant lymphoid cells 2 could be acting as linkers between the events happening in the skin and the responses carried out in distant MALT regions. Allergic patients tend to present higher levels of this cell type in their circulation, which supports this hypothesis, and it is in accordance with previous reports of peripheral ILC2s growing in number after intranasal challenge and during the pollinic season in patients with a cat and *Phleum pratense* allergy, respectively (99, 100). An increase in the number of ILC2s has been observed in diverse allergic diseases including FA, allergic rhinitis, asthma, and AD. Interestingly, ILCs express S1PR1, which regulates their egress from tissues to the blood, which could explain the higher number of ILCs found in inflammatory allergic conditions. As a proof of concept, patients treated with the S1PR1 agonist fingolimod (FTY720) show decreased numbers of circulating ILCs and less inflammatory ILC2s in the lungs (101).

Several studies have also confirmed the association of ILC2s with allergic asthma (102), a pathology where a CER/S1P imbalance has been related to uncontrolled inflammatory phenotypes (103). Increase ILC2s are also found in skin lesions from patients with AD, where the composition of ceramides is altered (104).

**Figure 3:**
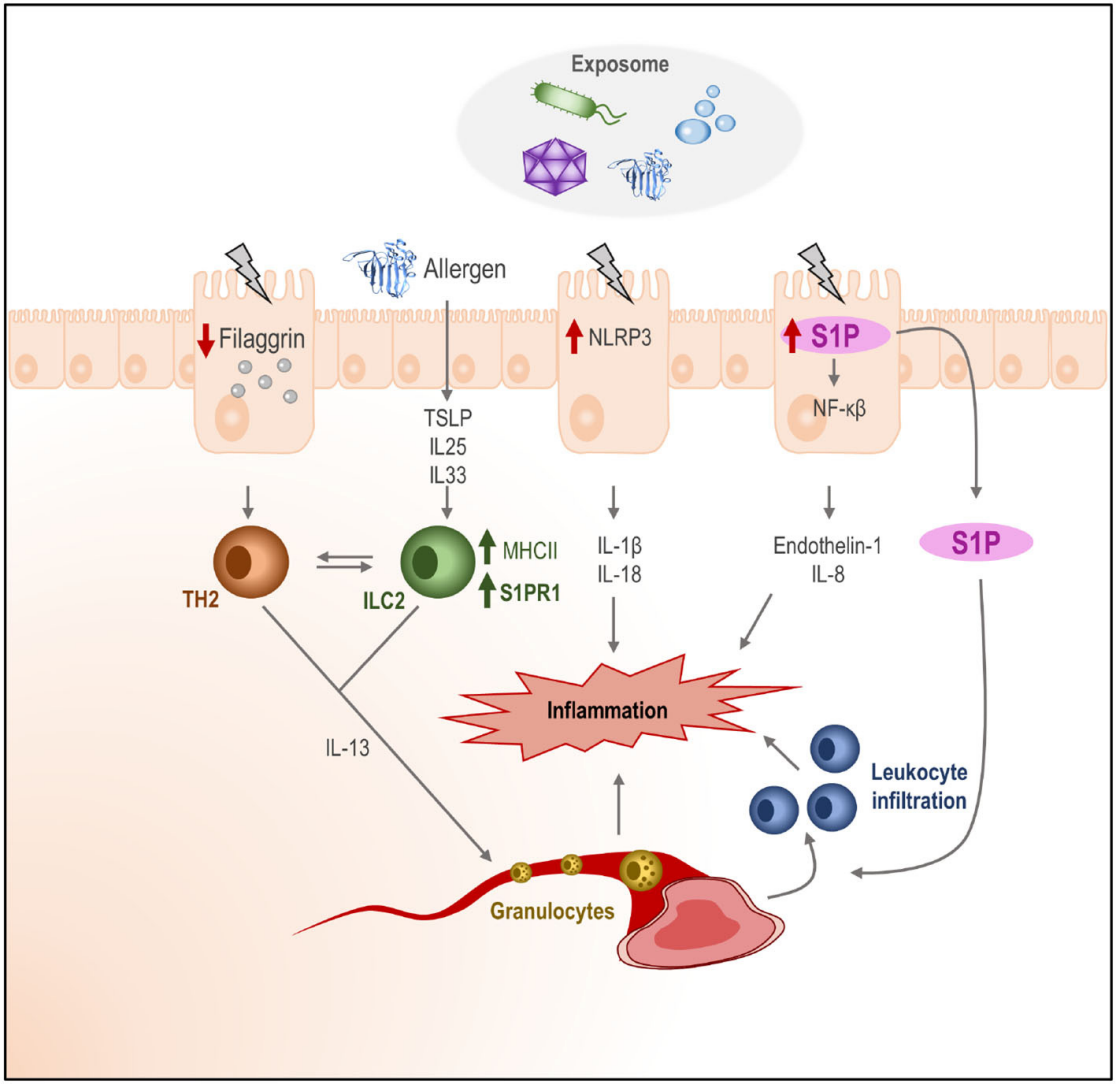
Effect of the exposome in human tissues and role of the S1P. The exposome (bacteria, allergens, chemicals, etc.) may cause damage in the outer layers of the skin. Keratinocytes may activate (depending on the nature of the signal) different intracellular routes for the activation of the inflammasome (NLRP3), the production of S1P, and the downregulation of filaggrin production, which would increase skin permeability. Additionally, alarmins (IL25, IL33, and TSLP) and pro-inflammatory cytokines (IL1b and IL18) would be produced by keratinocytes, initiating an immune activation state in the dermis, where ILC2 would be activated and their receptor pattern would change. The release of S1P to the dermis may increase leukocyte infiltration. The homed cells activated ILC2, and leukocytes in the cytokine context on the dermis would create an inflammatory state in the skin.

Therefore, the local response of the skin to an insult (due to any component of the environment or an exposome) would result in an increased permeability and the production of pro-inflammatory (IL1b, IL18) cytokine-like proteins (TSLP, IL25, and IL33). In the case of AD, due to a higher skin permeability, allergic sensitization is favored. In the context of an impaired skin barrier, the allergen or antigen would be more likely to gain access to the dermis, where most of the immune cells are found. Once there, ILC2 could be activated and acquire an antigen-presenting cell profile, as well as a migratory capacity, thanks to the cytokine environment. From the skin, they would relocate to other immune niches and activate the T2 response, although the details of how this activation would occur remain unknown (Figure 3).

What seems to be increasingly clear is that FA is the result of a long process of sensitization, followed by a phase of symptoms. Emerging evidence in recent years supports that both phases do not necessarily occur in the same place in the body. Increasing evidence supports the thesis that continued damage to the skin favors allergic sensitization, which then spreads throughout the body, favoring symptoms at distal sites of sensitization, such as the gastrointestinal tract.

### Sphingolipids and Anaphylaxis

Anaphylaxis is an acute life-threatening multisystem syndrome resulting from the sudden release of different cell mediators in the bloodstream (105). Among the cell types that contribute most to this condition are MCs, tissue-resident effectors that respond to diverse stimuli by releasing a wide variety of mediators such as histamine. The effects of these mediators range from local to systemic inflammation. One of the most well-known stimuli received by MCs is *via* the FcεRI (an IgE receptor), which activates the SphKs to produce S1P, which, in turn, induces MC degranulation.

**Figure 4:**
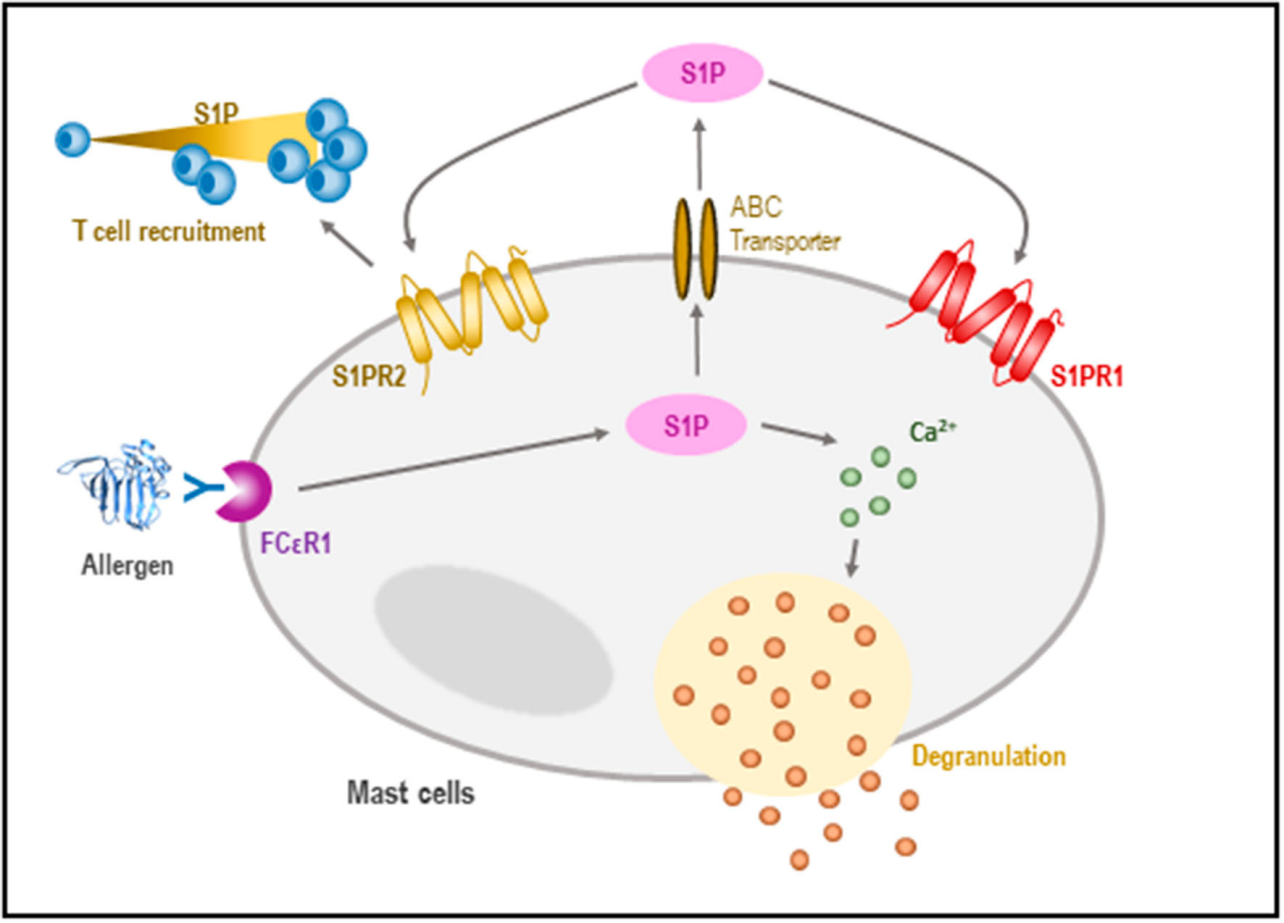
Role of S1P in the regulation of mast cells (MCs). In response to an antigen, the activation of SphK takes place, leading to the formation of S1P. S1P may influence calcium homeostasis, allowing MC degranulation. Likewise, S1P can be released outside the cell, where it is recognized by sphingosine receptors which promote both chemotaxis and T-cell recruitment.

Sphingosine-1-phosphate production by MCs contributes to the T2 response, which is key in the establishment of allergic inflammation. Evidence suggests that S1P has a direct effect on MCs acting in an autocrine manner *via* S1PR1 and S1PR2 (106). The binding of S1P to S1PR1 is translated into cytoskeletal rearrangements, regulating the migration of MCs toward antigens, while the activation of S1PR2 triggers MC degranulation (107, 108) (Figure 4).

In a model of systemic anaphylaxis, rapid depletion of plasma S1P to lower than 20% of basal levels was observed, contributing to cardiovascular shock during systemic inflammation. This suggests that S1P may be a potential diagnostic molecule in human disease (109).

Anaphylaxis is intimately related to endothelial disruption. In relation to the endothelial barrier, the junctions in the interendothelial cleft are covered by a layer of the fibrous matrix [the endothelial surface glycocalyx, which supports the maintenance of the vascular barrier and restricts the adhesion of leukocytes and platelets to the surface of endothelial cells (110)].

Sphingosine-1-phosphate-mediated stabilization of the endothelial surface can be achieved by reducing the action of matrix metalloproteinases, which degrade this layer. Similarly, in endothelial cells, Rac1 activation also regulates the intracellular localization of cortactin, where the actin-binding protein interacts with myosin light chain kinase to regulate actin dynamics during the S1P- and ATP-induced endothelial barrier strengthening (110). Moreover, S1P supports vascular integrity and vascular tone *via* S1PR1 and S1PR2; however, only the latter is crucial for survival (25).

The endothelial barrier homeostasis is regulated by S1P *via* S1PR1 and S1PR3 through Giα signaling, resulting in the induction of actin polymerization upon S1P stimulation of endothelial cells. Furthermore, the non-muscle myosin light chain kinase isoform gets phosphorylated, resulting in increased cell–cell adhesion (110).

## Conclusion and Remarks

Over the past years, advances in omics have led us to change the view on lipids: from seeing them as passive components of the cell barrier to understanding them as active components and mediators of processes on cellular and tissular levels. Among these lipids, sphingolipids belong to a special family with a wide variety of effector functions: maintaining epithelial integrity, helping immune cell differentiation and migration, acting as pro-inflammatory signals, and participating in cell proliferation/apoptosis homeostasis (for summary, Table 2). The metabolism of sphingolipids and their most studied products 1P and C1P is tightly controlled at enzymatic and binding receptor levels and their alterations contribute to the immune dysregulation that promotes the development of allergies. Sphingolipid molecules bound to allergens might thus have a relevant role in allergic sensitization. This increases the interest in studying them, not only to better understand the mechanisms underlying allergic diseases but also for their potential use as diagnostic and/or new prevention and treatment strategies to improve the lives of allergic patients.

**Table 2:** Role of sphingolipids in allergic diseases.

**Disease**	**Markers^**a**^**	**Role/process^**a**^**	**References**
Skin disease	↑S1P^b^ ↓CER ↑Filaggrin ↓C1P ↑ iNKTs	S1P: - Keratinocyte differentiation - ↑Permeability (↓tight junctions)	Danso et al. (78) Janssens et al. (70) Japtok et al. (64) Li et al. (79) Peters et al. (73) Peters et al. (73) Reines et al. (80)
Asthma	↑S1P in BALF ↑ORMDL3	S1P: - ↑Recruitment of alveolar macrophages (↓tight junctions) - ⊕ Mast cells, eosinophils and dendritic cells ORMDL3: - ⊗Sphingolipids - ↑ CER	Arana et al. (17) Cantero-Recasens et al. (46) Ghidoni et al. (37) Ha et al. (47) James et al. (19) Kawa et al. (42) Kowal et al. (5) Luthers et al. (45) Miller et al. (58) Ono et al. (6) Roviezzo et al. (39) Schauberger et al. (4) Yang and Uhlig (7) Zhai et al. (20)
Food allergy	S1P ↓CER	S1P: - ↑Recruitment and expansión of mast cells - ⊕ iNKTs - ⊕ ILCs - IgA CER: - ⊕ Desgranulation	Huang (111) Norris and Blesso (87) Nilsson (112)

a *↑,increase; ↓, decrease; ⊕, activation; ⊗, inhibition.*

b *S1P, Sphingosine-1-phosphate; CER, Ceramide; C1P, Ceramide- 1-phosphate; iNKTs, Invariant natural killer T cells; BALF, Bronchial alveolar liquid fluid; ILC, Innate lymphocyte cells*.

## Author Contributions

AD-P, ME, MG-A, DO, EI-A, JT-A, and DB have made a substantial, direct and intellectual contribution to the work. AD-P and DB wrote the final version of the manuscript. All authors contributed to the article and approved the submitted version.

## Conflict of Interest

The authors declare that the research was conducted in the absence of any commercial or financial relationships that could be construed as a potential conflict of interest.

## Abbreviations

AD: atopic dermatitis
C1P: ceramide-1-phosphate
CER: ceramide
CERK: ceramide kinase
ER: endoplasmic reticulum
FA: food allergy
ILCs: innate lymphoid cells
iNKTs: invariant natural killer T cells
MC: mast cells
SPH: sphingosine
SphK: sphingosine kinases
S1P: sphingosine-1-phosphate
S1PRs: sphingosine-1-phosphate receptors.
